# Non-Invasive Matrices for the Human Biomonitoring of PFAS: An Updated Review of the Scientific Literature

**DOI:** 10.3390/toxics13020134

**Published:** 2025-02-12

**Authors:** Martí Nadal, José L. Domingo

**Affiliations:** Laboratory of Toxicology and Environmental Health, School of Medicine, TecnATox, IISPV, Universitat Rovira i Virgili, Sant Llorenç 21, 43201 Reus, Catalonia, Spain; joseluis.domingo@urv.cat

**Keywords:** per- and polyfluoroalkyl substances (PFAS), human biomonitoring, urine, hair, nails, environmental exposure

## Abstract

Per- and polyfluoroalkyl substances (PFAS) are synthetic chemicals widely used in consumer and industrial products due to their unique physicochemical properties. However, their persistence and bioaccumulative potential pose significant environmental and human health risks. This review focuses on the use of non-invasive matrices—urine, hair, and nails—for the human biomonitoring of PFAS, highlighting key findings from scientific studies. While urine offers a non-invasive and practical option, its limited sensitivity for long-chain PFAS requires further analytical advances. Hair and nails have demonstrated potential for use in biomonitoring, with higher detection frequencies and concentrations for certain PFAS compared to urine. The variability in PFAS levels across studies reflects differences in population characteristics, exposure sources, and geographic regions. This review emphasizes the need for standardized analytical methods, expanded population studies, and the use of complementary matrices to enhance the accuracy and reliability of PFAS exposure assessment.

## 1. Introduction

Per- and polyfluoroalkyl substances (PFAS), formerly known simply as perfluorinated compounds (PFC), are defined as a class of chemicals with at least one aliphatic perfluorocarbon moiety [1,2]. PFAS are formed by a fully or partly fluorinated carbon chain (per- and poly-, respectively), which can be connected to different functional groups [2,3]. PFAS include a wide range of compounds that, due to their characteristics and properties (e.g., surfactants, friction reducers, and repellents of water, dirt, and oil), have been used in numerous applications over the past few decades [4,5,6]. Thus, PFAS have been used in textiles, electronics, food packaging, electric car batteries, and medical device manufacturing. Carpets, glass, paper, clothing and other textiles, plastic articles, cookware, electronics, as well as personal care products are examples of the numerous consumer products that contain or are coated with PFAS [4,5,7]. Unfortunately, most PFAS are non-degradable, or are finally transformed into stable terminal products [8,9]. The manufacturing of PFAS and their continued use over the years in numerous commercial and industrial applications has inevitably led to the passage of these substances into the environment, and consequently to human and ecological risks [6,10]. Exposure to PFAS in the general (non-occupationally exposed) population may mean substantial health risks on various systems and/or organs [11,12,13,14,15,16], while serious ecological risks have also been reported [17,18,19,20,21,22]. As a result, in recent years there has been a marked increase in efforts to reduce or to eliminate the production and commercial use of various PFAS through regulatory measures and bans [23,24,25].

In the current century, the understanding of the levels of human exposure to PFAS has been, and remains, a critical concern in environmental health. Similar to other environmental contaminants, one of the key techniques for assessing PFAS accumulation in humans is human biomonitoring (HBM). HBM involves measuring the levels of PFAS, or their breakdown products, present in an individual’s body [26]. Samples of invasive matrices (blood, internal tissues/organs) or non-invasive matrices (milk, urine, hair, nails) are usually collected [27]. Regarding PFAS, most HBM studies have been focused on the use of total blood/plasma/serum [28,29,30], as well as human milk [31,32], while other non-invasive matrices are less frequent. This review aims to summarize and discuss the findings of scientific studies where PFAS levels were measured in three non-invasive human matrices: urine, hair, and nails.

## 2. Search Strategy

The publications included in this review were cited in at least one of these two databases used for the current search: PubMed (https://pubmed.ncbi.nlm.nih.gov/) and Scopus (https://www.scopus.com), both accessed on 15 November 2024. There was no limitation on the search period. Combinations of the keywords perfluorinated compounds, PFC, perfluoroalkyl substances, polyfluoroalkyl substances, PFAS, urine, hair, nails, saliva, and non-invasive matrices were used in the search (e.g., PFC in hair, or PFAS in urine). Only articles published in English were included in this review. 

**Figure 1:**
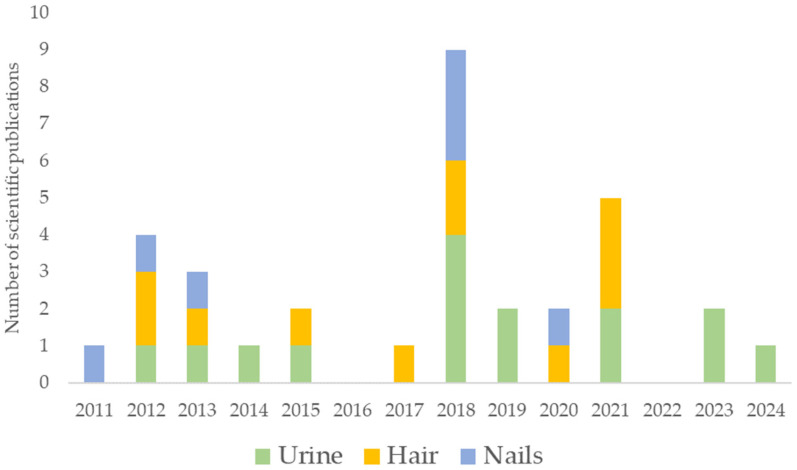
Scientific publications on PFAS levels in non-invasive human matrices in the period 2011–2024.

Among these three non-invasive matrices, human urine was identified as the most frequently used monitor, followed by human hair and nails. However, unlike other chemicals, there has not been a progressive increase in publications between 2011 and 2024. A summary of the number of publications and the matrix are depicted in Figure 1. Geographically, China leads the research on the biomonitoring of PFAS in non-invasive matrices. There is also available information in some European countries (e.g., Spain, Italy, Austria, and Belgium), while data from the US are limited and only restricted to human urine.

## 3. PFAS Levels in Non-Invasive Human Matrices

### 3.1. PFAS in Human Urine

Pérez et al. [33] developed and validated the first automated analytical method to measure PFC in samples of human hair and urine, which was based on turbulent flow chromatography and tandem mass spectrometry (TFC–LC–MS–MS). The presence of various PFC was determined in samples from donors living in Barcelona (Catalonia, Spain). In urine, 44% of the 18 targeted PFC were detected. PFBA (found in 100% of the analyzed samples, with a median value of 336 ng/mL), PFHxA, and PFOA were the most detected compounds. The median levels of PFHxA, PFHpA, PFOA, PFDA, PFUnDA, PFBS, and PFHxS in urine ranged between 39.4 ng/mL (PFHxA) and 0.66 ng/mL (PFOA), while the concentrations of PFPeA, PFNA, PFDoA, PFTrA, PFTeA, PFHxDA, PFODA, PFOS, PFDS, and PFOSA were below the respective limit of detection (LOD). In China, Li et al. [34] measured the concentrations of PFOA and PFOS in urine samples from 64 donors from the general population of Shanxi province. While PFOS was detected at a high frequency (95%), PFOA was found in only 56% of the samples. The mean (range) values of these PFC in urine were 12.9 (4.8–57.4) ng/L and 49.6 (<7.0–159.9) ng/L for PFOA and PFOS, respectively. In Republic of Korea, Kim et al. [35] conducted, for the first time in that country, a study aimed at measuring and comparing the concentrations of several PFC in paired serum and urine from children (from 5 to 13 years old) from Dae-gu. The concentrations in the urine of children were compared with those of Korean adults aged 20–29 years. Among the 16 analyzed PFC, the total concentrations in urine samples ranged between <LOD and 14.87 ng/mL and between <LOD and 7.60 ng/mL for children and adults, respectively. PFPeA, followed by PFHxA, and PFHpA were the predominant PFAS, with mean concentrations of 2.34, 0.73, and 1.35 ng/mL, respectively, in children, and 2.39, 1.38, and 0.49 ng/mL, respectively, in adults. The correlations between the levels of PFC in the serum and urine of children were assessed using individual and total levels. The concentrations of PFPeA, PFHxA, and PFHpA, as well as the total PFC levels in the urine and serum of children were compared. No significant relationships between the concentrations in both matrices were found. In a study conducted in China, Zhang et al. [36] measured the levels of PFOS and PFOA in 81 paired samples of whole blood and urine from adults in Tianjin, as well as in pregnant women. In urine samples from adults from the general population, the mean (range) concentrations—expressed in µg/g creatinine—of both PFAS were 0.031 (<LOQ-1.125) and 0.005 (<LOQ-0.020) for PFOS and PFOA, respectively. In turn, the levels of PFOS and PFOA in pregnant women were 0.015 (<LOQ-0.132) and 0.006 (<LOQ-0.030), respectively. When these results were compared with the levels of PFOS and PFOA in blood, it was noted that the concentrations in urine were three orders of magnitude lower than those found in the blood of both groups, namely, adults and pregnant women. 

At that time, methods capable of simultaneously quantifying PFAS, such as HBM programs, were unavailable. To address this gap, Kato et al. [37] developed an analytical method to measure these compounds in serum and urine. Their approach utilized on-line solid-phase extraction coupled with high-performance liquid chromatography and isotope dilution tandem mass spectrometry (on-line SPE-HPLC-MS/MS). In that study, 50 urine–serum paired samples (from individuals with no known exposure) were analyzed to validate the method. Among the 18 PFAS analyzed in the urine samples, only PFBA could be detected in 56% of the samples (median concentration: 0.2 ng/mL) at levels close to its LOD. According to the authors, those preliminary results suggested a very limited exposure to short-chain PFAS, as well as fluorinated alternatives, among the volunteers included in the study.

In Shenzhen (Southern China), Wang et al. [38] determined the levels of PFHxA, PFOA, PFNA, PFDA, PFUnDA, PFHxS, and PFOS in 39 matched human samples of serum, urine, hair, and nails. Regarding urine, the frequency of detection (DF) ranged between 5.13% for PFNA and 76.92% PFOS; the DF for PFOA was 33.33%. The urinary mean values of PFOA, PFNA, PFDA, PFUnDA, PFHxS, and PFOS were 4.61, 0.46, 0.39, 0.75, 6.91, and 18.71 ng/L, respectively. Based on these results, as well as those obtained with other biological matrices analyzed in that same study, it was concluded that urine would not be the best matrix for the biomonitoring of the most well-known PFAS, like PFOS. In the same line of research, Wang et al. [39] also carried out in China a survey aimed at investigating the use of three non-invasive matrices (hair, nails, and urine) for assessing human exposure to PFAS. Samples were collected from highly exposed fishery employees from Wuhan and a background exposed population of Shijiazhuang. Specifically, urine, PFBA, PFPeA, PFOA, PFBS, and PFHxS were found in all samples at relatively high concentrations, while PFNA, PFOS, and C8 Cl-PFESA were detected in more than 93% of the analyzed samples, but at lower concentrations. In exposed fishery employees, the following urine concentrations (ng/mL) of PFAS—given in decreasing order—were found: PFOS (6.03), PFBS (3.35), PFBA (1.43), PFPeA (1.02), PFHxS (0.32), and PFOA (0.16). The same PFAS were also detected in the urine samples of individuals from Shijiazhuang, but at much lower concentrations (units: ng/L instead of ng/mL), as follows: PFOS (4.93), PFBS (17.23), PFBA (19.52), PFPeA (19.38), PFHxS (16.38), and PFOA (20.91). Due to the lower detection levels, urine would not be the most appropriate monitor for the PFAS with ≥9 perfluorinated carbons. In the USA, Calafat et al. [40] measured the levels of 17 PFAS in samples of urine from 2682 participants of the 2013–2014 National Health and Nutrition Examination Survey (NHANES, USA). That survey was also focused on evaluating the usefulness of urine as a potential biological monitor of PFAS exposure in non-occupationally exposed subjects. Only a low percentage of the population showed detectable levels of PFAS in urine samples, but often the values were close to the LOD. The most frequently detected PFAS and their concentration ranges were as follows: PFHxA (27.2%; <LOD-7.5 μg/L), PFBA (13.3%; <LOD-3.4 μg/L), GenX (1.2%; <LOD-0.4 μg/L), and PFHpA (1.1%; <LOD-0.3 μg/L). Regarding PFOS, one of the PFAS for which—together with PFOA—the most data are available, the highest level in urine (0.6 μg/L) also corresponded to the highest level of this compound in serum (1270 μg/L), which was found in a previous study conducted by the same research group [37]. This outcome was in agreement with those of previous studies in which concentrations of long-chain PFAS were found to be much higher in serum than in urine. It was concluded that serum concentrations were the most reliable indicator for the biomonitoring of PFAS, regardless of their biopersistence. In Shanghai (China), Wu et al. [41] determined the urinary levels of 14 PFC in a very specific group of people: saleswomen from clothing shops. These people work in indoor environments for long periods, being exposed daily to a potentially considerable amount of textiles. In relation to this, it had been previously reported that dust and air (indoors) were factors that affected human exposure to PFC [42]. Thus, samples of indoor dust were collected and analyzed. The median urinary levels of PFC in saleswomen ranged between 10.15 and 666.1 ng/L for PFDS and PFOA, respectively. PFHxS, PFHxA, PFDA, PFUnDA, and PFTrDA were found in 100% of the samples, with the rest of the analyzed PFAS detected at concentrations >93%, except for PFHpA, whose DF was 62%. Among the analyzed perfluoroalkyl sulfonates (PFSAs), PFBS was the most predominant with a mean concentration of 109.6 ng/L, while that of PFOS was 23.58 ng/L. In turn, PFOA was the most predominant perfluoroalkyl carboxylate (PFCA) in urine, with a mean value of 662 ng/L. Although significant relationships were found between long-chain PFC in dust and urine, those investigators suggested that dust ingestion was a minor pathway, not representing a serious health concern regarding PFC exposure. In another occupational study on human exposure to PFAS, Peng et al. [43] measured the urinary concentrations of 17 PFAS in samples from 307 individuals working in acrylic fiber facilities and chemical plants. PFBA was by far the compound which showed the highest levels in the urine of workers from both the acrylic fiber plant and the chemical plant, with median values of 44.36 and 39.01 ng/mL, and DFs of 88.16% and 86.52%, respectively. PFBA, PFPeA, PFHxA, PFDoA, PFTrDA, PFTeDA, PFBS, and PFHxS were also detected in both groups, although at lower DFs and lower concentrations. The levels of the remaining analyzed PFAS, including PFOS and PFOA, were below the LOD.

In Hong Kong, Li et al. [44] measured the levels of seven PFAS in the samples of urine (and hair) from preschool children (4–6 years old), as well as in kindergarten air and water samples. With respect to the urine samples (53), these seven PFAS were detected at DFs higher than 50%, except for PFOS (32%), with PFOA and PFDDA showing DFs of 100%. The mean urinary concentration (ng/L) of the seven PFAS were, in decreasing order, as follows: PFDA, 5.09; PFOA, 4.66; PFOS, 4.22; PFHpA, 2.03; PFHxA, 1.04; PFDDA, 0.56; and PFNA, 0.42. While these PFAS concentrations were found to be rather low, higher concentrations of PFAS were detected in airborne particles sampled from kindergartens. Therefore, it was concluded that the health effects of low-dose but long-term PFAS exposure could not be overlooked. In Guangzhou (China), Peng et al. [45] measured the levels of several PFAS in the paired serum and urine samples (175) of waste recycling workers. Regarding the urine samples, among the 21 analyzed PFAS, 48% were found to be higher than the LOD. The highest DFs corresponded to PFBA and PFPeA (both 99%), while the lowest percentage (1%) corresponded to PFBS and PFOA. PFNA and PFUnDA were not detected in any sample. PFBA and PFPeA showed the highest urinary levels, with median values of 33.9 and 17.0 ng/mL, respectively. The median values of PFOS and PFOA, as well as the other 13 PFAS, were lower than the LOD, while that of the ∑PFAS was 57.5 ng/mL. In general, in that occupational study, PFAS concentrations were lower in serum than in urine samples. In Austria, Hartmann et al. [46] determined the concentrations of 33 xenobiotics in the urine samples of 85 healthy primary school children (aged 6–10 years). Nine out of the nineteen analyzed PFAS could be detected, with PFHxA and PFOA found in 100% of the samples. PFHpA, PFPeA, PFOS, and PFNA also showed high DFs: 99%, 96%, 78%, and 60%, respectively. One child had high urinary levels of PFPeA at 0.56 μg/L (0.98 μg/g of creatinine), and PFHxA at 0.61 μg/L (1.1 μg/g of creatinine), compared to the rest of participants. This was remarkable considering that the remaining 84 participants showed concentrations in urine samples of <0.040 μg/L for both substances. Among the detected PFAS, the maximum values (µg/g of creatinine) corresponded to PFHxA (0.61) and PFPeA (0.56), with 0.060 and 0.025 µg/g of creatinine being the maximum values for PFOA and PFOS, respectively. Based on these results, a recommendation of the authors was that because of the widespread exposure to PFAS, a restriction on all non-essential uses of these substances should be required. On the other hand, He et al. [47] recently reported the results of an investigation focused on assessing the potential association between the human urinary concentrations of PFAS and adverse effects on the kidney, which were basically based on studying urinary small-molecule metabolites. Seventy-two matched samples of urine and serum were collected from individuals working at a mega fluorochemical manufactory in Hubei province (China). Additionally, 153 samples of urine from local residents were also collected, and the levels of 23 PFAS were determined in both groups. The median (range) concentration of Σ_23_PFAS in the workers’ urine was 72.7 ng/mL (1.01–2124 ng/mL). In that study, PFHpA, PFOA, PFBS, PFPeS, PFHxS, PFHpS, PFOS, and 6:2 FTS were the eight selected PFAS for the model analysis and metabolomics analysis. All these eight compounds had DFs of 100%, except for 6:2 FTS (90.3%). The mean concentration of Σ_8_PFAS in urine was 141 ng/mL. In the control group, the urinary levels of PFAS were comparatively low, with 3.76 ng/mL being the median of Σ_23_PFAS and 1.73 ng/mL being the mean of Σ_8_PFAS. The concentrations of each PFAS in the control group were significantly lower than those found in the group of workers. The urine metabolomics study is out of the scope of the present review. However, we would like to highlight that according to the authors, association models showed positive correlations between the concentrations of PFAS in urine samples with markers of kidney function. This suggests that urine might be used to assess the potential adverse effects on the kidneys derived from high exposure levels of PFAS. In a recent study, also related to the adverse effects of PFAS in humans, Dai et al. [48] investigated the metabolic and transport characteristics of 21 PFAS in patients with liver diseases, while the PFAS differences between matched samples of blood/serum and the urine samples of patients from a hospital in Guangzhou (China) with and without hepatocellular carcinoma (HCC), were also compared. In urine samples, the highest DFs corresponded to PFBA (97%) and PFHxPA (92%), while PFOPA, PFDoA, and 8:2diPAP were not detected in any of the 180 analyzed samples. The DFs of PFOS and PFOA were 22% and 24%, respectively. PFPeA and PFBA showed the highest mean concentrations (3.72 and 2.11 ng/mL), respectively, with those of PFOS and PFOA being 0.022 ng/mL and <LOD, respectively. For the blood/serum and urine samples, the total PFAS concentrations in HCC patients were higher than in non-HCC subjects. A summary of the results from the above reviewed studies is provided in Table 1.

### 3.2. PFAS in Human Hair

The limited number of studies measuring PFAS levels in human hair are discussed next, while data are summarized in Table 2. In some studies, PFAS concentrations in other biological matrices from the same individuals, primarily serum, urine, and nails, were also determined. The urinary PFAS levels reported in these studies have already been discussed above and are therefore not reiterated in this section, while the levels in nails will be addressed below. Li et al. [49] developed methods to determine the concentrations of PFC in human hair and nail samples, which were subsequently applied to measure the levels of PFHxA, PFOA, PFNA, PFDA, PFUnDA, PFDoA, PFHxS, and PFOS in 15 samples of these matrices collected from the general population of Shanxi province in China. In the hair samples, the DFs of PFOA and PFOS were 100%, with concentration ranges of <LOQ-1.68 ng/g and <LOQ-6.74 ng/g, respectively. In contrast, PFHxA, PFDA, and PFDoA were not detected. In the study by Pérez et al. [33], in addition to the samples of urine, the levels of the same 21 PFCs were also determined in 24 samples of human hair. Although PFOS (range: 3.7–7 ng/g) and PFOA (range: 0.1–6 ng/g) were the most frequently detected PFC, the highest concentrations corresponded to PFBS (range: 6–39 ng/g), a compound that was found only in five samples, and PFHxS (range: 5.6–13.3), which was detected in four samples. In turn, PFPeA, PFHxA, PFHpA, PFUnDA, PFDoA, PFTrA, PFTeA, PFHxDA, PFODA, PFDS, FOSA, FHEA, and FOEA were below the LOD—or the LOQ—of the analytical method. While PFOS and PFOA were the most detected compounds in hair, the most predominant chemicals in urine were PFBA, PFOA, and PFHxS. The occurrence of both PFOS and PFOA in urine, hair, and nails were also examined by Li et al. [34], who analyzed the levels of these PFAS in matched samples of these non-invasive matrices collected from 64 donors from Shanxi province, China. As in urine, the mean level of PFOS in hair samples was higher than that of PFOA. Thus, the mean (range) concentrations of PFOS and PFOA in hair were 1.06 (0.08–6.74) ng/g, and 0.69 (<0.11–1.95) ng/g, respectively. In turn, Alves et al. [26] analyzed the concentrations of 15 PFAS in 30 samples of human hair from a non-exposed population from Antwerp, Belgium, by means of a novel miniaturized method using liquid chromatography tandem mass spectrometry (LC-MS/MS). Eight PFAS were detected in the hair samples, while PFBA, PFHxA, PFOA, PFBS, PFHxS, and PFDoS were quantified in >60% of the analyzed samples. The levels in hair of these compounds ranged between 0.014 and 1.53 ng/g, with the average values ranging from 0.046 to 0.214 ng/g. These values were notably lower than those usually found in invasive matrices, such as serum and blood, or non-invasive monitors, like urine.

Kim and Oh [50] also developed and validated an analytical method to determine the levels of PFAS in human hair, using LC-MS/MS and four different extraction procedures to optimize the extraction method. Hair samples were collected from 47 volunteers in Busan Metropolitan City, Republic of Korea, in which 11 PFAS were monitored. From these eleven PFAS, nine compounds were found at different DFs, with six PFAS being <30%, while PFBS and PFHpS were not detected in any sample. The highest levels corresponded to PFHxS, PFOA, and PFHxA, with mean levels of 3.27, 1.32, and 1.09 ng/g, respectively, while PFOS was detected in only two samples (mean: 1.03 ng/g). Wang et al. [38,39] measured the concentrations of various PFAS (PFOA, PFNA, PFDA, PFUnDA, PFHxS, and PFOS) in 39 matched samples of serum, urine, hair, and nails from the general population of Shenzhen (China). In hair, PFOS and PFOA were detected at relatively high DFs of 92.31% and 71.79%, with mean (range) values of 0.60 (<0.03–1.60) ng/g and 0.25 (<0.03–0.96) ng/g, respectively. In contrast, PFHxS was not detected in any hair sample. Among the analyzed PFAS, PFOS was the most common in the four matrices examined in that survey [38]. In another study conducted by the same research group, Wang et al. [39] evaluated the feasibility of using non-invasive matrices, such as hair, nails, and urine, to assess internal exposure to PFAS. In addition to the samples of a group of highly exposed fishery employees from Tangxun Lake, China, samples from residents of Shijiazhuang were also collected. The concentrations of 17 PFAS were measured in 41 hair samples. C8 Cl-PFESA, PFOS, PFHxS, and PFOA had the highest DFs, with percentages of 88%, 83%, 71%, and 68%, respectively. However, the mean concentrations of PFAS in hair followed a different order, as follows: PFOA (5.62 ng/g), C8 Cl-PFESA (1.13 ng/g), and PFOS and PFHxA (both 0.60 ng/g). To conclude, the authors highlighted that the concentrations of C8 Cl-PFESA and PFOS found in hair reflected internal exposure to these PFAS.

The concentrations of seven PFAS in the hair samples of 53 preschool children in Hong Kong were determined by Li et al. [44], who also measured the levels of these PFAS in the urine samples of those children (age: 4–6 years). PFOS and PFDDA were not detected in any sample, while the DFs and mean values of the remaining five analyzed PFAS were as follows: PFOA, 63%, 0.248 ng/g; PFHpA, 59%, 0.028 ng/g; PFHxA, 70%, 0.021 ng/g; PFNA, 70%, 0.007 ng/g; and PFDA, 48%, 0.005 ng/g. Although the results of that study, which also included measurements of these same PFAS in urine samples, suggest that health risks for these children may not be a concern, as previously mentioned, the effects of long-term exposure to low doses of PFAS should not be overlooked. To measure the levels of PFAS in the hair samples of Italian individuals, Piva et al. [51] developed and validated a new LC-MS method of PFAS analysis in 11 samples from volunteers, based on accurate mass measurements (Quadrupole Time-of-Flight, QTOF). Twenty PFAS were analyzed, but only PFBA, PFBS, PFOA, and PFOS could be quantified, with PFOS and PFOA being the most frequently detected compounds. The ranges of the concentrations of these compounds were between <LOQ (PFOS) and 0.587 ng/g (PFNA), except for a high level of PFBA (14.58 ng/g) which was found in the sample of a subject who lived in a zone widely contaminated by PFAS. In a subsequent study carried out by the same researchers [52], the concentrations of 20 PFAS were measured in the hair samples of 86 Italian subjects living in the regions of Veneto, Lombardy, Emilia-Romagna, and Marche. PFAS were not detectable in 15% of the samples, while in an additional 18.6%, the PFAS levels were below the LOQ. In the hair samples of the rest of the participants (66.4%), at least one PFAS could be quantified. PFOA and PFOS, with mean concentrations of 0.1402 and 0.1155 ng/g, respectively, had the highest DFs, followed by PFBA, PFNA, PFDA, PFUnA, and PFHxS. Relatively high levels were also found for PFBA and PFNA, with mean values of 0.376 and 0.120 ng/g, respectively. On average, the mean sum of PFAS was 0.146 ng/g (range 0–0.85 ng/g). As could be expected, the results of that study showed notable differences in the presence of PFAS in hair depending on the of the population.

### 3.3. PFAS in Human Nails

Liu et al. [54] conducted a study aimed at assessing the levels of seven PFAS in human fingernails and toenails as a potential biomarker of exposure to these compounds. Samples of nails and blood were obtained from 28 healthy volunteers (age: 20−50 years) from Dalian University of Technology (China). PFOS, PFNA, PFDA, PFDoA, and PFTA were found in all samples of both fingernails and toenails, while PFHxS was not detected in any sample. PFOS showed the highest concentrations, with median values of 33.5 and 26.1 ng/g for fingernails and toenails, respectively. These values were followed by those corresponding to PFNA, with median levels in fingernails and toenails of 20.4 and 16.8 ng/g, respectively. PFOA, PFDA, PFDoA, and PFTA showed median concentrations between 0.19 and 8.94 ng/g. Considering also the values of the PFAS found in serum, the authors concluded that the levels of PFAS in nails showed a good correlation with the concentrations in blood, suggesting the potential utility of nails as a biomarker for PFAS. 

In addition to hair samples, Li et al. [49] measured the levels of eight PFC in samples of human (from the general population of Shanxi province, China) nails. In contrast with the results found in hair samples, the eight analyzed PFAS were detected in all nail samples, with PFOA, PFOS, and PFHxS having DFs of 100%. The concentrations (ranges, ng/g) of these compounds were <LOQ-0.43, 0.15–5.09, and 0.36–2.79, respectively. In a subsequent study conducted by the same research group [34], matched samples of urine, hair, nails, and urine were collected from 64 donors from Shanxi province, and the levels of PFOA and PFOS were determined. In nails, the DFs and mean (range) concentrations were 94% and 0.24 (<0.14–0.56) ng/g and 97% and 1.04 (0.15–5.09) ng/g for PFOA and PFOS, respectively. Based on the results obtained in urine, hair, and nails, the authors suggested that nails could have more potential than urine and hair for the HBM of PFOA and PFOS in non-occupationally exposed individuals. Wang et al. [38,39] carried out a couple of studies aimed at determining the occurrence and levels of PFOA, PFNA, PFDA, PFUnDA, PFHxS, and PFOS in 39 matched samples of serum, urine, hair, and nails from a non-exposed population of Shenzhen (China). In the nail samples, PFOS had a DF of 87.18%, somewhat less than the DF found in hair (92.31%), but slightly higher than the DF in urine samples (76.92%). In turn, PFOA was detected in only 17.95% of nail samples, in contrast to 71.79% in hair, and 33.33% in urine samples. On the other hand, the DFs of PFNA, PFDA, and PFUnDA in nails were much higher than those for hair and urine. The mean concentrations of PFNA, PFUnDA, PFHxS, and PFOS in nails were 0.27, 0.23, 0.44, and 0.69 ng/g, respectively, while those of PFOA and PFDA were <014 and <0.24 ng/g, respectively. It was concluded that nails could be better than other non-invasive matrices for determining PFAS in general, but especially for PFOS biomonitoring [38]. In a subsequent survey whose characteristics were described above [39], only seven of the seventeen analyzed PFAS could be detected and/or quantified. The DFs (in parentheses) were as follows: PFOA (100%), PFHxS (98%), PFOS (98%), C8 Cl-PFESA (95%), PFBS (85%), PFNA (80%), and C10 Cl-PFESA (56%). The highest concentrations corresponded to PFOA, 29.18 ng/g; PFOA, 3.89 ng/g; and C8 Cl-PFESA, 3.65 ng/g. According to the authors, the validity of nail samples for measuring internal exposure to PFAS could vary depending on the analyzed PFAS, as well as the specific populations.

The last study on this topic found in PubMed and/or Scopus was carried out by Liu et al. [53], who measured the levels of PFAS in human serum, hair, and nails from non-occupationally exposed volunteers living in ten cities in Guangdong province (southern China). Regarding nails, the concentrations of 11 PFAS were determined in 39 samples. The DF of PFOS for nail samples was 100%, while high DFs were found for PFHxS (98%) and PFOA (91%), while those of PFHpA (63%), PFPeA (58%), and PFNA (42%) were lower. In contrast, PFHxA, PFDA, and long-chain PFAAs (C ≥ 11) were not detected. The mean concentrations of the PFAS showing the highest occurrence in nails were as follows: PFOS, 33 ng/g, a level that meant a contribution of 47% to ΣPFAAs; PFHpA, 12 ng/g; PFHxS, 9.3 ng/g; PFPeA, 8.5 ng/g; PFOA, 5.4 ng/g; and PFNA, 2.2 ng/g. Interestingly, the levels of PFOS and PFOA in nails were significantly higher than those found in hair, while in contrast, those of PFHxS were significantly higher in hair than in nails. No significant differences were observed among the levels of other PFAS between hair and nails. Table 3 summarizes the findings of the studies above-mentioned.

At the end of the last decade, Jian et al. [55] published a short review focused on human exposure to PFAS and tissue (blood, urine, milk, hair, and nails) distribution. These authors observed that the distribution of PFAS in urine was influenced by the chain length of these compounds and human gender. Although data on PFAS in hair and nails certainly remained limited, they indicated that these matrices showed potential as non-invasive tools for assessing human exposure to PFAS.

## 4. Discussion

The present article was aimed at reviewing the results of studies carried out to determine the concentrations of PFAS in human samples of the non-invasive matrices of urine, hair, and nails. Although saliva is another non-invasive matrix that has also been used for HBM in occupational and environmental studies [56,57,58,59,60], to the best of our knowledge, no scientific paper has reported—to date—the concentrations of PFAS in human saliva.

The studies examined in the present review highlight the variability in PFAS concentrations in human urine across different populations, geographical locations, and levels of occupational exposure. The results of the studies reviewed here are consistent with the growing body of evidence suggesting that urinary PFAS concentrations are generally lower than those found in blood or plasma/serum, reflecting the limited excretion of these compounds via urine. Although the development of advanced analytical methods has notably improved the ability to detect PFAS in urine at relatively low concentrations, analytical challenges still remain for the detection of many PFAS, particularly those with long chains. This underscores the interest in and importance of the continued refinement of analytical techniques to accurately quantify PFAS in HBM studies. The above reviewed studies show that urinary PFAS levels vary significantly among populations, reflecting differences in environmental exposure, lifestyle, and regulatory measures. For example, in studies conducted in China (e.g., Shenzhen, Wuhan), higher urinary PFAS levels were observed among individuals occupationally exposed to fluorochemicals or living near industrial areas, while studies conducted in the USA and Europe reported lower PFAS levels in the urine samples of general (non-occupationally exposed) populations, with PFBA, PFHxA, and PFHpA being frequently detected at low concentrations. On the other hand, studies performed in Republic of Korea and Austria showed that children often have higher urinary PFAS concentrations than adults, which might potentially be due to differences in metabolism or exposure pathways.

Urine is a non-invasive biomonitoring matrix, making it an attractive option for assessing human PFAS exposure [46,61,62]. However, its utility is still limited by the low detectability of long-chain PFAS. Studies comparing urine and serum concentrations consistently report higher levels in serum, suggesting that this is a more reliable biomonitor for PFAS. On the other hand, occupational exposure studies, such as those involving chemical plant workers, reveal significantly higher urinary PFAS levels compared to non-occupationally exposed populations. Moreover, emerging research links urinary PFAS concentrations to potential health effects, including kidney function markers and associations with liver diseases like hepatocellular carcinoma (HCC). These findings emphasize the need for targeted biomonitoring and risk assessment in high-exposure groups.

On the other hand, the analysis of PFAS in human hair and nails as non-invasive matrices has revealed significant insights into their potential for biomonitoring. However, the data remain sparse, and the results across studies demonstrate variability depending on the compounds analyzed, the population characteristics, and the analytical methods used. Hair seems to be a viable matrix for assessing PFAS exposure, with numerous studies detecting key compounds like PFOA, PFOS, and PFHxS. Detection frequencies for these compounds often exceeded 70%, highlighting their consistent presence in human hair. The mean concentrations vary across studies, with PFOS and PFOA frequently observed at higher levels than other PFAS. For example, Wang et al. [38,39] and Li et al. [34] reported mean PFOS concentrations of 0.60–1.06 ng/g, with PFOA concentrations typically being lower but still notable. Geographical variations were also quite evident across data sets. Thus, studies from China, such as those by Wang et al. [38,39], showed higher mean PFOS and PFOA concentrations than those conducted in Europe, such as Alves et al. [26], where the average PFAS levels were lower (0.046–0.214 ng/g). This could reflect regional differences in PFAS exposure sources, regulatory policies, and environmental contamination. Moreover, PFBS and PFHxS were occasionally reported at higher concentrations, particularly in the study by Pérez et al. [33]. These findings indicate that while PFOS and PFOA remain dominant, other PFAS compounds like PFBS can also reach significant levels in specific populations.

In turn, nails have also been identified as another promising matrix for PFAS biomonitoring, showing—in some cases—potential for higher DFs and concentrations compared to hair. Thus, Li et al. [49] demonstrated that PFOS and PFOA were consistently detected in nails, often at higher concentrations than in hair, while Liu et al. [53] found mean PFOS levels of 33 ng/g and PFOA concentrations of 5.4 ng/g in nails, significantly exceeding hair concentrations from the same population. The variability in DFs and concentrations across studies highlights the complexity of using nails as a biomonitoring tool. While compounds like PFHxS and PFOS exhibited high detection rates (>90%) in some studies, others showed lower occurrences for long-chain PFAS. Regional contamination and individual differences, such as age, gender, and lifestyle factors, likely contribute to these discrepancies.

The comparison between hair and nails underscores the complementary nature of these matrices. Hair seems to be more sensitive to compounds like PFHxS, while nails tend to accumulate higher levels of PFOS and PFOA. This suggests that the combined analysis of both matrices could provide a more comprehensive understanding of internal PFAS exposure. In relation to this, Wang et al. [38,39] and Liu et al. [53] demonstrated that nails might be particularly effective for monitoring PFOS, given the higher DFs and concentrations in that matrix.

Beyond the potential usefulness of these non-invasive human matrices, in recent years, some alternatives, which could be considered as minimally invasive, have been developed. Volumetric absorptive microsamplers (VAMSs) are a promising technique to overcome some of the main limitations of PFAS analyses in human blood, especially in children’s exposome studies [63,64].

## 5. Conclusions

While urine is a valuable biomonitoring tool for short-chain PFAS and occupational studies, its limitations for the exposure assessment of long-chain PFAS in the general population require complementary approaches. Further investigations should prioritize enhancing analytical capabilities, understanding health effects, and implementing preventive measures to mitigate PFAS exposure and its associated risks. On the other hand, hair and nails are also valuable non-invasive options for the HBM of PFAS. The studies reviewed here confirm the ability of these matrices to reflect internal exposure, with PFOS and PFOA consistently emerging as key analytes. Nails are particularly promising monitors for the detection of PFAS with high molecular weights, while hair could be more sensitive to short-chain compounds.

Further research should focus on standardizing analytical techniques to improve comparability, including the combined use of non-invasive and minimally invasive matrices, such as VAMSs. Furthermore, new investigations should be targeted on diversifying populations and geographic regions, and investigating the correlations between PFAS levels in different biomonitoring matrices to identify the optimal markers for human exposure and health risk assessments. Anyway, the implementation of stricter regulations to limit non-essential uses of PFAS, particularly in regions with high exposure levels, is essential.

## Figures and Tables

**Table 1:** Summary of PFAS studies in human urine.

Location	Study Population	Most Detected PFAS	Concentration Range	Reference and Publication Year
Barcelona, Spain	General population (*n* = 30)	PFBA (100%)	<LOQ-2890 ng/L (PFOA);	Pérez et al. [33], 2012
Shanxi, China	General population (*n* = 63)	PFOS (95%)	4.8–57.4 ng/L (PFOA); <7.0–159.9 ng/L (PFOS)	Li et al. [34], 2013
Dae-gu, Republic of Korea	Children (5–13 y) and adults (20–29 years)	PFPeA	Children: <LOD-14.87 ng/mL; Adults: <LOD-7.60 ng/mL	Kim et al. [35], 2014
Tianjin, China	Adults and pregnant women (*n* = 81)	PFOS	Adults: <LOQ-1.125 µg/g creatinine (PFOS); Pregnant women: <LOQ-0.132 µg/g creatinine (PFOS)	Zhang et al. [36], 2015
USA	General population (*n* = 50)	PFBA (56%)	0.2 ng/mL (median)	Kato et al. [37], 2018
Shenzhen, China	General population (*n* = 39)	PFOS (76.92%)	18.71 ng/L (mean PFOS)	Wang et al. [38], 2018
Shijiazhuang, China	General population (*n* = 41)	PFBA, PFOA (98%)	<LOQ-64.37 ng/mL (PFOA); <LOQ-19.26 ng/mL (PFOS)	Wang et al. [39], 2018
Wuhan, China	Exposed fishery employees (*n* = 8)	Multiple (100%)	0.04–0.31 ng/mL (PFOA); 1.13–16.54 ng/mL (PFOS)	Wang et al. [39], 2018
USA	NHANES participants (*n* = 2682)	PFHxA (27.2%)	<LOD-7.5 μg/L (PFHxA)	Calafat et al. [40], 2019
Shanghai, China	Clothing shop saleswomen	Multiple (100%)	207.2–906.7 ng/L (PFOA); 0.73–150.7 ng/L (PFOS)	Wu et al. [41], 2019
Hong Kong	Preschool children (4–6 years) (*n* = 53)	PFOA, PFDDA (100%)	0.45–28.1 ng/L (PFOA); 1.39–33.6 ng/L (PFOS)	Li et al. [44], 2021
China	Workers of a chemical plant and an acrylic fiber plant (*n* = 307)	PFBS (>86%)	<LOD-15.70 ng/mL (PFBS)	Peng et al. [43], 2021
Austria	School children aged 6–10 years (*n* = 85)	PFHxA, PFOA (100%)	0.00065–0.060 μg/L (PFOA); <LOD-0.025 μg/L (PFOS)	Hartmann et al. [46], 2023
Hubei, China	Workers (n = 72) and residents (*n* = 153)	Multiple (100%)	Workers: 1.01–2124 ng/mL; Residents: 3.76 ng/mL (median)	He et al. [47], 2023
Guangzhou, China	Hospital patients (*n* = 180)	PFBA (97%)	<LOD-0.627 ng/mL (PFOA); <LOD-0.530 ng/mL (PFOS)	Dai et al. [48], 2024

**Table 2:** Summary of PFAS studies in human hair.

Location	Study Population	Most Detected PFAS	Concentration Range	Reference and Publication Year
Shanxi, China	General population (*n* = 15)	PFOA, PFOS (100%)	<LOQ-1.68 ng/g (PFOA); <LOQ-6.74 ng/g (PFOS)	Li et al. [49], 2012
Shanxi, China	General population (*n* = 53)	PFOA (98%)	<0.11–1.95 ng/g (PFOA); 0.08–6.74 ng/g (PFOS)	Li et al. [34], 2013
Barcelona, Spain	General population (*n* = 24)	PFOS (46%)	<LOQ-6.1 ng/g (PFOA); <LOQ-7 ng/g (PFOS)	Pérez et al. [33], 2012
Antwerp, Belgium	General population (*n* = 30)	PFBS (100%)	<LOQ-74 ng/g (PFOA); 34–120 ng/g (PFBS)	Alves et al. [26], 2015
Busan, Republic of Korea	General population (*n* = 47)	PFOA (87%)	<LOD-3.12 ng/g (PFOA); <LOD-1.16 ng/g (PFOS)	Kim and Oh [50], 2017
Shenzhen, China	General population (*n* = 39)	PFOS (92%)	<0.03–0.96 ng/g (PFOA); <0.03–1.60 ng/g (PFOS)	Wang et al. [38], 2018
Shijiazhuang, China	General population (*n* = 41)	C8 Cl- PFESA (88%)	<LOQ-51.07 ng/g (PFOA); <LOQ-2.65 ng/g (PFOS)	Wang et al. [39], 2018
Hong Kong	Preschool children (4–6 years) (*n* = 27)	PFHpA, PFNA (70%)	105–459 pg/g (PFOA)	Li et al. [44], 2021
Italy	General population (*n* = 11)	PFOS (55%)	<LOD-0.178 ng/g (PFOA); <LOD-0.239 ng/g (PFOS)	Piva et al. [51], 2021
Four regions in Italy	General population (*n* = 86)	PFOA, PFOS (39%)	0.08–0.25 ng/g (PFOA); 0.08–0.239 ng/g (PFOS)	Piva et al. [52], 2021
Guangdong, China	General population (*n* = 39)	PFOS (100%)	<0.44–13 ng/g (PFOA); 2.3–67 ng/g (PFOS)	Liu et al. [53], 2020

**Table 3:** Summary of PFAS studies in human nails.

Location	Study Population	Most Detected PFAS	Concentration Range	Reference and Publication Year
Dalian, China	University students and professors (*n* = 28)	Multiple (100%)	Fingernails: <0.38–16.4 ng/g (PFOA); 1.41–165 ng/g (PFOS) Toenails: <0.38–9.54 ng/g (PFOA); 4.15–153 ng/g (PFOS)	Liu et al. [54], 2011
Shanxi, China	General population (*n* = 15)	PFOA, PFOS, PFHxS (100%)	<LOQ-0.43 ng/g (PFOA); 0.15–5.09 ng/g (PFOS)	Li et al. [49], 2012
Shanxi, China	General population (*n* = 63)	PFOS (97%)	<0.14–0.56 ng/g (PFOA); 0.15–5.09 ng/g (PFOS)	Li et al. [34], 2013
Shenzhen, China	General population (*n* = 39)	PFUnDA (92%)	<0.04–0.46 ng/g (PFOA); <0.05–1.89 ng/g (PFOS)	Wang et al. [38], 2018
Shijiazhuang, China	General population (*n* = 41)	PFOA (100%)	0.77–29.18 ng/g (PFOA); <LOQ-3.89 ng/g (PFOS)	Wang et al. [39], 2018
Wuhan, China	Exposed fishery employees (*n* = 8)	Multiple (100%)	<0.18–1.34 ng/g (PFOA); <57.38–479.58 ng/g (PFOS)	Wang et al. [39], 2018
Guangdong, China	General population (*n* = 39)	PFOS (100%)	<0.44–19 ng/g (PFOA); 10–45 ng/g (PFOS)	Liu et al. [53], 2020

## Data Availability

Data will be made available on request.

## Abbreviations

The following abbreviations are used in this manuscript:

PFAS	per- and polyfluoroalkyl substances
PFC	perfluorinated compounds
HBM	human biomonitoring
PFBA	perfluorobutanoic acid
PFPeA	perfluoropentanoic acid
PFHxA	perfluorohexanoic acid
PFHpA	perfluoroheptanoic acid
PFOA	perfluorooctanoic acid
PFNA	perfluorononanoic acid
PFDA	perfluorodecanoic acid
PFUnDA	perfluoroundecanoic acid
PFDoA	perfluorododecanoic acid
PFDDA	perfluoro-1,10-decanedicarboxylic acid
PFTrA	perfluorotridecanoic acid
PFTeA	perfluorotetradecanoic acid
PFHxDA	perfluorohexadecanoic acid
PFODA	perfluorooctadecanoic acid
PFBS	perfluorobutanesulphonate
PFHxS	perfluorohexanesulphonate
PFOS	perfluorooctanesulphonate
PFDS	perflurodecanesulphonate
PFOSA	perfluorooctane sulfonamide
PFECA	perfluoroalkyl ether carboxylic acid
PFESA	perfluoroalkyl ether sulfonic acid
Cl-PFESA	chlorinated perfluoroalkyl ether sulfonic acid
PFDDA	perfluoro-1,10-decanedicarboxylic acid
LOD	limit of detection
LOQ	limit of quantification
DF	detection frequency
TFC–LC–MS–MS	turbulent flow chromatography and tandem mass spectrometry
SPE-HPLC-MS/MS	solid-phase extraction coupled with high-performance liquid chromatography and isotope dilution tandem mass spectrometry
QTOF	Quadrupole Time-of-Flight
HCC	hepatocellular carcinoma
